# Clinical laboratory services for primary healthcare centers in urban cities: a pilot ACO model of ten primary healthcare centers

**DOI:** 10.1186/s12875-021-01449-1

**Published:** 2021-05-27

**Authors:** Soha A. Tashkandi, Ali Alenezi, Ismail Bakhsh, Abdullah AlJuryyan, Zahir H AlShehry, Saeed AlRashdi, Maryjane Guzman, Marvin Pono, Franklin Lim, April Rose Tabudlong, Lamees Elwan, Musa Fagih, Ahmad Aboabat

**Affiliations:** 1 Pathology and Clinical Laboratory Medicine Administration (PCLMA), King Fahad Medical City, Second Central Healthcare Cluster (C2), Riyadh, Kingdom of Saudi Arabia; 2 Associate Executive Administration of Community Health (AEACH), Second Central Healthcare Cluster (C2), Riyadh, Kingdom of Saudi Arabia

**Keywords:** Primary Healthcare, Clinical Laboratory, ACO, Centralization, Laboratory Services, Laboratory Utilization, Quality, Key Performance Indicators, KPI

## Abstract

**Background:**

Primary healthcare centers (PHC) ensure that patients receive comprehensive care from promotion and prevention to treatment, rehabilitation, and palliative care in a familiar environment. It is designed to provide first-contact, continuous, comprehensive, and coordinated patient care that will help achieve equity in the specialty healthcare system. The healthcare in Saudi Arabia is undergoing transformation to Accountable Care Organizations (ACO) model. In order for the Kingdom of Saudi Arabia (KSA) to achieve its transformational goals in healthcare, the improvement of PHCs’ quality and utilization is crucial. An integral part of this service is the laboratory services.

**Methods:**

This paper presents a pilot model for the laboratory services of PHC's in urban cities. The method was based on the FOCUS-PDCA quality improvement method focusing on the pre-analytical phase of the laboratory testing as well as the Saudi Central Board for Accreditation of Healthcare Institutes (CBAHI) gap analysis and readiness within the ten piloted primary healthcare centers.

**Results:**

The Gap analysis, revealed in-consistency in the practice, lead to lower the quality of the service, which was seen in the low performance of the chosen key performance indicators (KPI's) (high rejection rates, lower turn-around times (TAT) for test results) and also in the competency of the staff. Following executing the interventions, and by using some of the ACO Laboratory strategies; the KPI rates were improved, and our results exceeded the targets that we have set to reach during the first year. Also introducing the electronic connectivity improved the TAT KPI and made many of the processes leaner.

**Conclusions:**

Our results revealed that the centralization of PHC's laboratory service to an accredited reference laboratory and implementing the national accreditation standards improved the testing process and lowered the cost, for the mass majority of the routine laboratory testing. Moreover, the model shed the light on how crucial the pre-analytical phase for laboratory quality improvement process, its effect on cost reduction, and the importance of staff competency and utilization.

## Background

Healthcare transformation is a global move, across countries and across the healthcare disciplines (medical, nursing, laboratory, etc.) [1–3], the move globally is towards becoming an accountable Care Organization (ACO). With ongoing technological and industrial developments in healthcare, coupled with the market-driven behaviors of the patients seeking medical care, there is now an urgent need to find the most cost-effective healthcare system/model without compromising the quality of care and patient safety. [4] This raises many questions about the effectiveness of these developments on patient care and healthcare transformation efforts, and whether these technologies are market-driven or patient-driven, or whether patients are in need of healthcare services or are consumers, and how the current patient care model impacted patients, families, overall population, and governmental expenses. Answers to the aforementioned questions are consequential to the patient’s expectations (demand) and on the services provided (supply) in the global health industry, regardless of a country’s level of income [1, 4].

The Saudi Ministry of Health (MOH) provides around over 60% of the healthcare services while the rest are shared among other government agencies (for example, hospitals operated by other ministries including the ministry of education, defense, national guard and security forces) as well as the private sector medical institutes [5]. Around 80% of the healthcare services provided by MOH and other governmental sectors is provided free of charge to the eligible service beneficiaries [5]. The Kingdom has made huge positive developments in the infrastructure and organization of its health care system, which was positively reflected on the life of its residents, for example; the strategic step of the national children immunization program against infectious diseases, another example is the national newborn screening program for inborn errors of metabolism. The country also introduced the Primary Health Care (PHC) concept as a basic healthcare delivery system in 1978. All of these development in the health care services has positively changed the health map of KSA [5]. From a financial prospective the MOH, is funded from the total government budget, and with the efforts to improve the services and expand to reach the residents in urban as well as rural areas, a significant increase in the allocated annual budget, has been witnessed reaching for example 7% of the government total budget in 2016, comparing to the 5.9% allocated in 2006 [6]. In spite of these efforts, and similarly to other public funded healthcare systems, the healthcare system in KSA is facing challenges that needed to be addressed for future viability and sustainability [5, 6]. Therefore, the healthcare sector transformation strategy addresses these challenges.

The Kingdom of Saudi Arabia (KSA) presented in the Ministry of Health (MOH) has stated in its "Health Sector Transformation Strategy", third volume (V3) eight challenges that face the Saudi healthcare system and need to be in the focus of its transformation initiatives. Among these challenges is the primary care being inadequate and inconsistent, which puts more load on the secondary and tertiary hospitals, and their associated resources [7]. Moreover, the document highlighted significant gaps in the quality & consistency of the services provided to patients, with a lack of consistent protocols and pathways for treatment, as well as measurement of outcomes on patients. The Saudi Central Board for Accreditation of Healthcare Institutions (CBAHI) Essential Safety Requirements Survey has also emphasized key deficits in safety across all categories of hospitals including the primary healthcare centers [7].

### Important Role of the Primary Care Centers in Healthcare

The Declaration of Alma Ata, which was adopted at the International Conference on Primary Health Care in 1978, was the first international declaration underlining the importance of primary healthcare. The primary healthcare approach has since been accepted by members of the World Health Organization (WHO) as the key to achieving the goal of *health for all* in developing countries. This initiative was extended to all WHO countries 5 years later when primary healthcare was identified as vital for maintaining *health for all* around the globe [8]. This created a fundamental need for the primary healthcare system to lead in the elevation of population health by providing appropriate acute responses to diseases or outbreaks, disease prevention, and end-of-life hospice care [4].

The WHO defines primary healthcare as a whole-of-society approach to health and well-being by focusing on the needs and preferences of individuals, families, and communities. It addresses the broader determinants of health and focuses on the comprehensive and interrelated aspects of physical, mental, and social health and well-being in order to provide whole-person care for health needs throughout a person’s lifespan. Primary healthcare ensures that people receive comprehensive care, ranging from promotion and prevention to treatment, rehabilitation, and palliative care in a familiar environment (https://www.who.int/primary-health/en/). It is designed to be first-contact, continuous, comprehensive, and coordinated patient care, which, if well implemented, will achieve equity in the specialty healthcare system [9]. Alhamdan et al. (2015) described the primary health center (PHC) as the basic structural and functional unit of the public health services, which provides an accessible and affordable healthcare system as well as preventive healthcare, such as screenings and management of chronic diseases. In other words, the PHCs are the backbone of any healthcare system [10]. De Maeseneer et al. (2008) stated that there are 12 characteristics that define effective primary healthcare services. They include general scope, accessibility, integration, and continuity and are based on multidisciplinary, holistic, personal, familial, communal, well-coordinated, confidential, and advocative teams [4]. In the De Maeseneer paper, six global challenges that face the primary healthcare system were defined:

1. The relevance of a general approach in the era of sub-specialized medical care.
2. The effect of cultural diversity on the accessibility of care.
3. Integration is perceived as a challenge to the current fragmentation in services, which are market-driven [11].
4. The meaning of continuous care in a time of changing demography caused by migration, war, and increased quality of life opportunities.
5. The effect of family-oriented care at a time where the nuclear family is being replaced by individuality.
6. Having a coordinated care that ensures that the patient is getting the right services, at the right time and manner with no duplication or medical error is a challenge in a time where the best practices is based on "single disease management".

### The Laboratory Role in the Transformation to ACO

The Clinical Laboratory Improvement Amendments (CLIA) defines the clinical and medical laboratory as any facility that performs laboratory testing on specimens obtained from humans to provide information for health assessment and for the diagnosis, prevention, or treatment of disease.

Accountable Care Organizations (ACOs) are a care delivery system that is based on the provision of coordinated high-quality and cost-effective care, valuing the quality of care over the volume [12]. Five strategies have been created to assist laboratory services in reaching their full potentials at an ACO institute. The first strategy is the capacity of outreach services and programs to extend beyond their institute. Second, is the electronic connectivity enables physicians to easily request and access laboratory tests they need. Third, is the establishment of lean processes will lead to better time utilization with less errors and an expanded capacity. Fourth, is the utilization management plays a crucial role in decreasing costs without jeopardizing the quality. Finally, laboratories must align with a bigger picture, where laboratory data plays an important role clinically and for ACO planning [3, 12]. The CLIA, through its resources and accreditation scheme, provides both the framework and resources for the laboratories to function maintaining best utilization, improve clinical outcome and become more efficient and effectiveness, which is the core of the ACO model and the targeted goal of the ACO proposed laboratory strategies [13].

### The Saudi National Healthcare Transformation Plan

Globally, the aging population is rising, from approximately 9.2% over the age of 60 in 1990 to 11.7% in 2013. This number is expected to reach 21.1% by 2050. In Saudi Arabia, the population is considered youthful with only approximately 5.2% being over the age of 60. This number is expected to reach 8.1% by 2025 and 21.8% by the year 2050 [10].

**Fig. 1:**
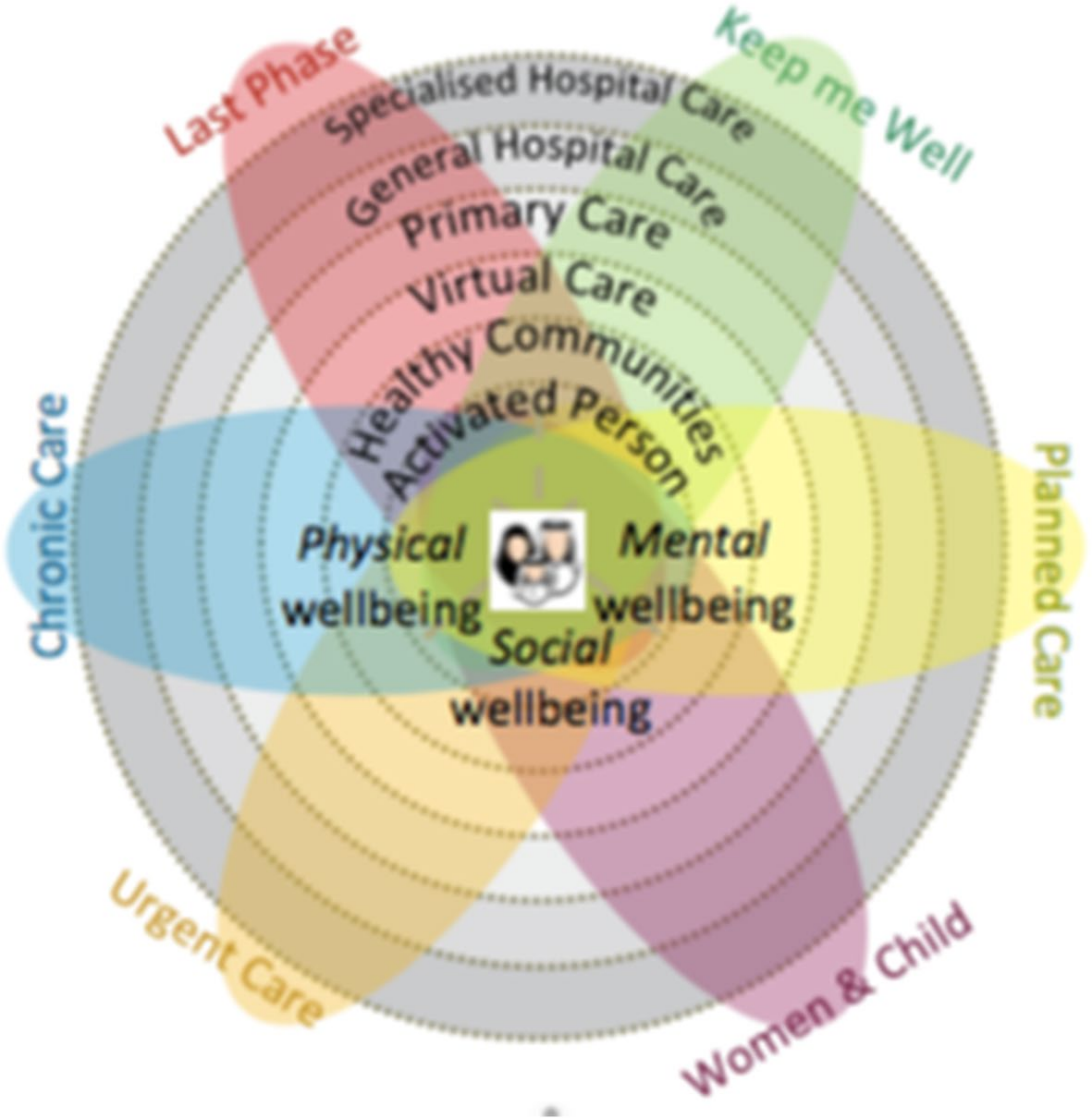
The new six-axes model of care for the 2030 vision of transforming healthcare in KSA

Saudi Arabia launched the National Transformation Program 2018–2020 (NTP), which is the operational program for the kingdom’s 2030 vision. This vision contains various strategic objectives, performance indicators, and commitments that are shared between public and private organizations in order to achieve excellence in government performance, economic development, and upgraded living services. The NTP has 8 dimensions with 37 objectives that contain a total of 433 initiatives. The elevation of healthcare services is the first of the eight dimensions [7]. The first dimension of NTP aims to restructure the health system to include a six-axes model of care (Fig. 1) for an integrated health system that strengthens population health through accessibility, community awareness, and disease prevention [7].

The reformation of the healthcare structure started with governmental institutes combining into healthcare clusters. Those clusters have been named according to geographical regions. Currently, few clusters have been established: Western Region (W1), Central Region (C1, C2, and C3), and Eastern Region (E1). Each cluster is composed of a reference hospital (tertiary hospital), 2 – 4 secondary hospitals, and a number of PHC (50 – 100) depending on the geographic catchment area. The second central healthcare cluster (C2) is based in Riyadh (the capital of KSA), it includes a medical city; King Fahad Medical City (KFMC) as the tertiary specialized reference institute, three other hospitals: Prince Mohammad Bin Abdulaziz Hospital, Al Yamamah Hospital, King Saud Chest Hospital, Dental centers, Renal Dialysis Centre, and a total of 56 PHC's. Each of the PHC's has a set number of clinics, radiology centers, pharmacies, and a laboratory.

The elevation of healthcare services stated three strategic objectives to be achieved through 70 initiatives (NTP, Arabic version, visited April 2019). Most of the initiatives for the transformation of healthcare will deliver into health services integration, multidisciplinary approaches, screening programs, prevention, population health, and patient safety, all of which the medical laboratories play an integral role. Having an established laboratory in each PHC may not be cost-effective, especially in less-populated areas; therefore, one suggestion is that diagnostic facilities should be made available to nearby hospitals, and another suggestion is to have referral PHCs with all diagnostic facilities [14, 15].

### The role of Saudi Central Board for Accreditation of Health Institutes (CBAHI) in Harmonizing the Healthcare services

The accreditation for healthcare institutions involves an accrediting body that surveys and verifies compliance with the recognized criteria and standards. The CBAHI is a nonprofit organization and is the only national agency authorized to accredit governmental and private healthcare institutes in Saudi Arabia. The main function of the CBAHI is to determine healthcare quality and patient safety standards for all healthcare facilities [16]. The assessment system assesses compliance with the institute by how well the facility fulfills standards, the implementation of standards, and the real practice of such rather than bulk document reviews [16]. For laboratories under CBAHI standards, there are a total of 11 standards (56 substandard) covering the expected laboratory functions for diagnosis, treatment monitoring, evaluation, and plans for future decisions. Most of the published studies related to CBAHI standards implementation and outcomes in PHCs were focusing mainly on patient satisfaction outcomes, which is an important quality improvement tool to measure the patient experience journey [17].

The scope of this paper is to present a piloted model of laboratory services implemented in ten PHCs within the C2 cluster. In this model, CBAHI assessment was conducted for the laboratory services provided by the 10 PHC's, the deficiencies were defined and interventions were made by applying some of the laboratory ACO strategies. The improvements following interventions were measured through a set of initial standard key performance indicators that are commonly used by laboratory standards to assess the quality of services provided. To our knowledge, this is the first study describing a laboratory model of service in KSA and shows the result of combing the implantation of some of the known ACO strategies with the CBAHI standards implantation and shows the outcomes.

## Methods

### Goal Alignment with the NTP Delivery Plan 2018–2020

**Fig. 2:**
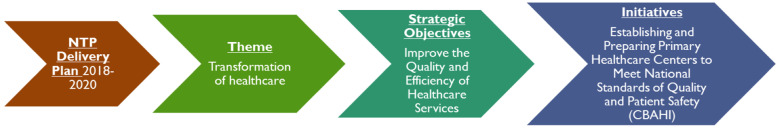
The National Transformation Program (NTP) initiative for PHC. This figure illustrates the main initiative which this pilot project is based on, and the strategic objectives of the transformation of healthcare theme it belongs to

This pilot study focused on aligning the laboratory model of service with the NTP 2018–2020 delivery plan (Fig. 2) through improvement of the quality of the PHC's Laboratories services and utilization of their resources to prepare them for the CBAHI inspection, which is one of the KSA healthcare NTP initiatives. This also assessed how by implementation of the ACO laboratory strategies this can improve the overall PHC laboratory quality & utilization, which will positively impact their performance. We have used the national CBAHI PHC standards to assess PHC laboratory services and define the baseline quality of the laboratory services provided by each of the ten PHC's. The study took place over 13 months, from February 8, 2018, to February 28, 2019. The first 3 months included data collection, document reviews, assessment of practice based on the CBAHI standards for PHC, and gap analysis to determine the baseline of each PHC laboratory in terms of quality of service and utilization of resources. This was followed by intervening corrective actions to standardize the practice, implement quality, utilize management programs, and improve laboratory services.

### FOCUS-PDCA Approach

A team from the KFMC laboratory was formed to assess the PHC laboratories. The team consisted of three CBAHI laboratory assessors, a safety officer, a laboratory technician from the central receiving section who assessed specimen handling, a point of care testing (POCT) technician, and a phlebotomist.

In order to define and standardize the quality status of the laboratory, a FOCUS-PDCA approach was followed in three phases: baseline assessment, defined, and executed interventions, assessment of results, and conclusions of the model of service. FOCUS-PDCA is an acronym meaning: Find, Organize, Clarify, Understand, Select, Plan, Do, Check, and Act, this strategy is used for process improvement. The PDCA approach was first presented by Dr. Edwards Deming, quality management expert in the 1950′s. Opportunities for improvements were defined according to the level of compliance with the PHC’s laboratory CBAHI standards assessment results. Standards were marked as not met, partially met, or minimally met and were considered opportunities for improvement.

The assessment visits were planned to be a minimum of two visits per PHC. The initial visit aimed to determine the PHC baseline quality level, and the follow-up visit assessed the results of interventions. For the baseline assessment, the centralization of laboratory testing to the KFMC Pathology and Clinical Laboratory Medicine Administration (PCLMA) began. Key performance indicators (KPIs) (KPI definitions and targets are defined later in this paper) were defined to reflect the quality and staff competency in specimen collection, labeling, handling, and transportation.

Interventions were determined according to the initial results (presented in the results section). However, interventions covered three main areas:

1. Staff competency, which included standardized (on-site and off-site) training of nurses on both phlebotomy techniques, POCT techniques, and policy and procedural awareness
2. Document control system, which included the generation of unified policies and procedures, as per the clinical scope of the ten PHC's
3. Quality and safety program, which is a standardized model of laboratory services and continuous quality control

### CBAHI Standards Evidence of Compliance Approach

The first step was to ensure the eligibility criteria for the Saudi Central Board for Accreditation of Healthcare Institutes (CBAHI) inspection. This meant that providers’ healthcare services must be covered by CBAHI PHC accreditation standards, hold a current license, be in operation for at least 6 months prior to conducting the on-site survey, and had completed an application form.

The second step was to conduct an internal assessment for the lab section within each PHC using the CBAHI PHC standards guide (Version 3, 2017) to assess the evidence of compliances (EC), which were the on-site elements scored by the surveyor and were summarized in Table 1 [18]. Each EC was scored on a four-point scale:

- 3 = Fully met: ≥ 75% compliance with the EC for at least 4 months prior to the initial survey or 1 year for the triennial survey
- 2 = Partially met: ≥ 50% to < 75% compliance with the EC or compliance for at least 3 months prior to the initial survey or 9 months for the triennial survey
- 1 = Minimally met: ≥ 25% to < 50% compliance with the EC or compliance for 2 months prior to the initial survey or 6 months for the triennial survey
- 0 = Not met: < 25% compliance with the EC or compliance for less than 2 months prior to the initial survey or less than 3 months for the triennial survey
- NA = Not applicable, indicating that the standard or EC does not apply to the PHC

**Table 1:** CBAHI Standards and Evidence of Compliance. This table summarizes the 11 CBAHI standards that are under the Laboratory (LB) Chapter for primary healthcare centers presented with their evidence of compliance (EC) and method of inspection. For simplicity, the 56 sub-standard were not included, however they can be found by refereeing to the original document CBAHI PHC standards guide (Version 3, 2017)

Standard #			
LB.1	Laboratory services (LB) are available to meet patient needs, applicable to national standards		
	LB.1.EC.2	There is a written agreement with an accredited lab for the provision of special procedures and consultations	Document Review
LB.2	A current laboratory policies and procedures manual are readily available to staff. Policy and procedure manual should be well structured		
	LB.2.EC.1	There is evidence of comprehensive, approved, and current policies and procedures manual that are available and well known to the staff	Staff Interview
LB.3	The laboratory organization structure is defined and available		
	LB.3.EC.1	There is an updated and approved laboratory organization structure with sections and staff categories identified under the director supervision	Document Review
	LB.3.EC.2	Laboratory director is a qualified pathologist or a qualified clinical scientist	Personnel file
LB.4	The laboratory space is adequate for its function, well-maintained, free of clutter and does not compromise the quality of work and personnel safety		
	LB.4.EC.1	There is adequate lab space, that must have: two sinks with one sink used exclusively for handwashing, machines attached directly to a wall socket, critical machines attached to the emergency socket, adequate control of temperature and humidity, and telephone facility	Observation
LB.5	The laboratory establishes a documented safety program under the supervision of the laboratory director and consistent with the facility’s safety guidelines		
	LB.5.EC.1	There are fire and safety training records	Document Review
	LB.5.EC.2	There is an effective system for reporting and investigating occupational injuries and accidents	Document Review
	LB.5.EC.3	There is evidence of comprehensive, approved, and current safety manual that is available and well known to the staff	Staff Interview
	LB.5.EC.4	There are sufficient safety signs posted where appropriate	Observation
	LB.5.EC.5	Eye wash stations and emergency showers are available and checked at regular intervals	Observation
LB.6	The laboratory implements all the rules and guidelines of infection control		
	LB.6.EC.1	There are records to support the immune status or vaccination for all lab personnel	Personnel file
	LB.6.EC.2	Personnel protective equipment are available and used when appropriate	Observation
	LB.6.EC.3	There is evidence of the implementation of policies on universal precautions and prohibition of eating and drinking in the lab	Observation
	LB.6.EC.5	There is evidence of negative pressure monitoring in microbiology	Observation
	LB.6.EC.6	There is evidence of clear designation of clean and contaminated areas	Observation
LB.7	The laboratory publishes and distributes clear written instructions for proper collection, handling, transportation, and preparation of specimens		
	LB.7.EC.1	There is a laboratory specimen guide (LB.7.1-LB.7.7) distributed to all clinical departments	Observation
LB.8	The laboratory keeps instrument and equipment in proper functional condition through the establishment of a system where equipment are properly operated, cleaned, quality controlled, monitored and maintained		
	LB.8.EC.1	Inspection and preventive maintenance records for all laboratory equipment are maintained	Document Review
LB.9	Reagents and solutions are properly labeled, as applicable and appropriate		
	LB.9.EC.1	There are written policies and procedures for reagent preparation, labeling, storage, and expiration	Document Review
	LB.9.EC.2	Reagents are labeled in accordance with the laboratory policy	Observation
LB.10	The laboratory has a clear system for results reporting		
	LB.10.EC.1	There are written policies and procedures for reporting panic values (critical results)	Document Review
	LB.10.EC.2	There is evidence of that TAT for all laboratory services is defined, communicated, and agreed upon by clinical departments	Staff Interview
	LB.10.EC.3	There are records in support of proper reporting of panic values	Observation
LB.11	The laboratory must have a quality management program approved by the laboratory director and available for all laboratory personnel. The laboratory quality management program must be integrated with the center-wide quality program		
	LB.11.EC.1	There is a written quality management program satisfying all of the elements above	Document Review
	LB.11.EC.2	There is evidence of participation in external and/or internal proficiency testing program covering all laboratory analytes	Document Review
	LB.11.EC.3	There is evidence of using an efficient accident and adverse event reporting and investigating system	Document Review
	LB.11.EC.4	There is evidence of corrective and/or preventive measures taken when expected quality monitoring outcomes are not achieved	Document Review

In general, a PHC survey outcome will fall under one of the following categories:

- Accredited: none of the Essential Safety Requirement (ESR) standards are scored, the total score is no less than 85% compliance for ESR standards, and no ESR standards.
- Conditional accreditation: 5–1 ESR standards have been scored at zero, or the total score is between 75 and 85% in ESR standards, or no ESR standards.
- Denied accreditation: > 5 ESR standards have been scored zero, or the total score is < 75% in ESR standards, or no ESR standards.

### KPIs and Staff Competency

Monitoring of the PHCs’ laboratory performance was based on a selected set of universal KPIs set by the CLIA, which cover the three defined phases of laboratory processes: pre-analytical, analytical, and post-analytical.

The KPI choices were based on the rationale of building a unified infrastructure for quality monitoring at the staff level to ensure proper specimen collection and handling as this is a crucial element for the success of the total quality program of the C2 cluster of PHCs. The values and rates of KPI's that were presented in this manuscript were calculated following the KFMC Laboratory policies and procedures that were implemented and followed in-house. The Laboratory of KFMC is an accredited nationally by CBAHI and internationally by the college of American Pathologists (CAP), under each KPI in the following section, the equations from the KFMC polices that is used to calculate the percentage of the KPI is demonstrated.

Initially, three principal KPIs were selected for monitoring:

- Rejection rate: This KPI reflected the pre-analytical stage done at each of the PHC’s test requests, sample collection and handling, and transportation, until arrival at the reference laboratory. Many factors could contribute to sample rejection. Depending on the reason for rejection, a weakness within the pre-analytical process was determined, and a proper corrective action could be taken. The rejection rate was calculated as a percentage where the numerator and denominator are the total number of samples rejected from all the PHC's and the total number of samples received, respectively.
- Turn-around Time (TAT): This KPI reflected the analytical phase and defined the duration of time from specimen reception to result reporting. This KPI reflected the KFMC laboratory performance. The TAT rate was calculated as a percentage where the numerator and denominator are the total number of samples resulted on-time from to the PHC's and the total number of samples accepted by the KFMC laboratory, respectively.
- Amendment of results: This KPI reflected the post-analytical phase and defined the change of the result after reporting and informing the treating physician or caregiver. This KPI reflected the KFMC laboratory performance. The Amendment of results rate was calculated as a percentage where the numerator and denominator are the total number of results that were changed after the release of the final result to the PHC and the total number of samples resulted by the KFMC laboratory, respectively.

Table 2 represents the international benchmark for the selected KPIs and our first-yeartargets.

**Table 2:** First-year KPI targets and benchmark. This table represents the selected KPI's to be monitored during the first year, their benchmark, and the target set to reach for the first year of this pilot project

Phase	KPI	Benchmark	First-year target
Pre-analytical	Rejection rate	0.6%	7%
Analytical	TAT	> 90%	80%
Post-analytical	Amendment of result	0.1%	> 1%

#### Staff competency

Two main competency programs for the PHC nurses and laboratory personnel were designed to standardize laboratory practices within the PHC. The first program was for specimen handling and transportation, which was directed to laboratory personnel. The second program was divided into two parts. The first part included the laboratory test request and sample collection. This was directed to nurses within the PHC. The second part involved POCT testing competency and was directed to nurses.

All personnel, including drivers transporting the samples, were trained on laboratory safety measures related to their tasks, such as spill management. Competencies were then tested and secured. The programs covered the known six competency areas mandatory for annual assessment for the technical laboratory team [18–20]. They included the following: (1) direct observations of routine work (as per defined scope), (2) record monitoring, (3) procedural knowledge, (4) direct observations of instrument maintenance and function checks, (5) assessment of POCT test performance, and (6) assessment of critical thinking and problem-solving skills.

### The Cost of Quality

Here, two elements were addressed: the cost of suboptimum quality in the pre-analytical phase and the utilization of staff time.

The pre-analytical cost per sample was calculated in averages as follows: pre-analytical cost = average consumables + phlebotomy time + laboratory personnel time + transportation. The rejection rate cost improvement was measured by taking the rate reached in the month with the highest rejection rate and then comparing it with the first month of the second year, which was the month with the lowest rejection rate. The improvement percentage was then calculated.

Concerning the staff utilization element, laboratory personnel did lab tasks only when there were sample collections and transportation. Some of the piloted centers collected samples once a week (based on the workload and population), while others collected samples daily. For a general estimation of staff utilization, the following calculation was based on an average of three collections per week or 12 collections per month. Each session was approximately 3 h and had to comply with the predetermined collection and transportation schedule for each PHC.

## Results

At the start of this pilot study, the ten PHCs that had joining the C2 Cluster were already in operation. Pathology and Clinical Laboratory Medicine Administration at KFMC immediately started receiving samples from each of them on the bases of pre-study agreements, where a sample collection scheduling system had been determined with the stakeholders (the Executive Administration of Community Health, PHC directors, and the Executive Administration of the Associate Medical Administrations (AEAMA), represented by the PCLMA team). However, following the facility management and safety inspections done by other quality and safety teams from KFMC (results not shown), some centers were temporarily suspended from functioning and underwent renovation during the months following the commencement of this pilot study (March, September, and November).

### CBAHI Standards Evidence of Compliance Approach

Nine out of the ten PHCs were functional and underwent the initial assessment for PHC laboratory CBAHI standards (Version 3, 2017). The 11 laboratory standards and various sub-standards covered the major quality essentials for providing patient laboratory services. This included the physical structure, comprehensive safety program, staff competency, laboratory service model (including the specimen handling, transportation, and testing guide), equipment management program, reagent management program, document control system, and the total laboratory quality management program.

Most standards were found to be either not met or partially met:

- The CBAHI standards for PHC assessing the physical structure (standard LB.4) states that “the laboratory space is adequate for its function, well-maintained, free of clutter, and does not compromise the quality of work or personnel safety”. It also emphasizes adequate facilities, such as water taps, sinks, and drains, as well as an adequate electrical infrastructure, including an emergency power supply and climate control. The designated room for the laboratory within the PHC centers was designed and created in accordance with the national CBAHI standard. Two major findings were related to space adequacy. When implementing full laboratory services, a separate area for clean versus dirty laboratory activities was not possible, and the limited electrical infrastructure had no emergency ports to support the present equipment in the case of an electrical power interruption.
- The safety programs (standards LB.5 and LB.6) were found to be partially met for three out of nine sub standards. These included the consistent availability of disposal containers for the sharp consumables, underreporting of occupational incidents, and the absence of eye washing stations. Non-applicable sub standards were found at two out of nine PHCs, and the presence of fume hoods and emergency showers was not applicable as the types of lab services provided at the time of inspection did not mandate the need for this safety equipment. The LB.6 standard was concerned with the infection control program and contained seven sub standards. One out of the seven sub standards was not applicable as it related to the negative pressure facility for highly infectious material. These major findings showed nonconsistency in the personal protective supply and the lack of clearly designated areas for clean versus dirty activities.
- The quality control monitor and quality management program were reflected in compliance with standards LB.3 and LB.11. Standard LB.3 has three sub standards and is concerned with the laboratory organizational structure. This standard was not met. Standard LB.11 addressed the quality management program with a focus on KPI monitoring, proficiency testing scheme, incident reporting, and all related corrective and preventive actions. This standard was not met in any of the nine PHCs.
- Standards LB.1, LB.7, and LB.10 address the service provided. LB.1 has three sub standards that were found to be partially met. One of the PHCs provided some laboratory services, while the remaining centers utilized their laboratory tests. However, there was no written agreement or menu of tests provided. LB.7 addresses the specimen collection, handling, and transportation guidelines, which was found to be not met in all nine PHCs. The PHCs lacked an electronic system for sample requests and labeling (or barcoding). Instead, this was done manually using the patient name and file number, which in some cases was a single file number for an entire family file, meaning that there was more than one member under the family file. Moreover, LB.11, which addressed the TAT in reporting and the critical result reporting algorithm, was also not met.
- Standards LB.8 and LB.9 addressed the equipment management and reagent handling and inventory management, respectively, and both standards were found to be partially met in all inspected PHCs. The major finding for the equipment management standard was a lack of consistency in regular checks and record maintenance for some of the equipment. Concerning the reagent management standard, the documentation was again missing, had an inconsistent delivery schedule, and a clearly defined inventory with its monitor.
- Standard LB.2 addressed the document control system and was also not met due to outdated policies and procedures as well as a lack of existing documentation, such as service agreements, organizational structure for the laboratory, quality, and safety programs, personnel files, documented training activities, staff competency, and a service menu with a collection manual.

### Continuous Monitoring KPIs

Monitoring of PHCs’ laboratory performance was based on a set of universal KPIs set by the CLIA, covering the three defined phases of laboratory processes: pre-analytical, analytical, and post-analytical. For the first 8 months, from February to September 2018, the test requests, sample accessioning, result reporting, and rejection reporting were done manually for all ten PHCs. Starting from October 2018, implementation of the LIS system used in KFMC started at a rate of one PHC per month (implementation, staff training, and launch). By February 2019, four of the seven operating PHCs had the LIS system implemented and functioning.

#### The Rejection Rate

**Fig. 3:**
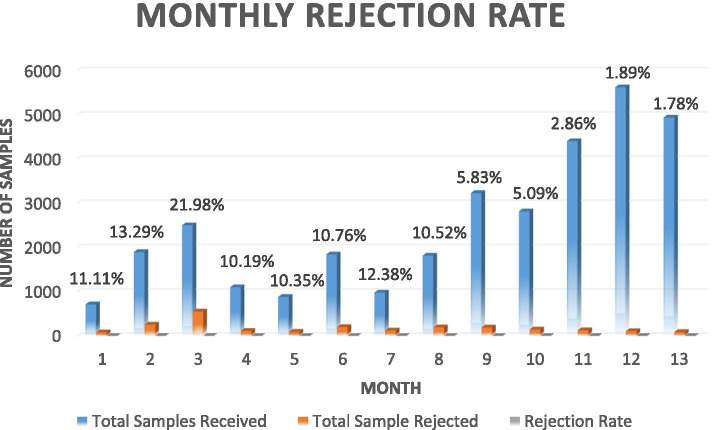
Monthly rejection rate in relation to the monthly samples received from the PHCs. The blue bar represents the total number of samples received at each month, the orange bar represents the total number of the rejected samples at receiving, and the last grey bar is the percentage (rate) of rejection for each month, the rate is stated at the top of the bars for each month for clarity

**Fig. 4:**
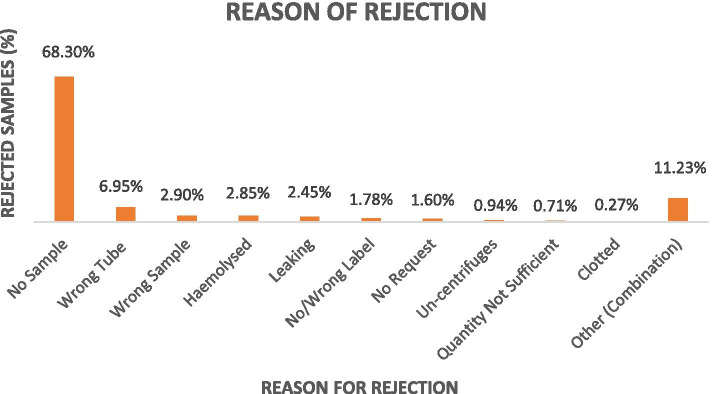
Percentages of rejected samples in relation to the reason for rejection

The rejection rate was monitored on a daily basis and was immediately communicated to each PHC (manager or representative) via email in order to arrange for a rapid repeat sample and avoid patient decision delays. Overall, rejection rates are presented in Fig. 3 and the correlation to the possible underlying reasons and proposed interventions were presented in Table 3. Of the rejected samples, the reasons for rejection included the following: request without a sample (68.30%), incorrect tube (6.95%), incorrect sample (2.90%), hemolysis (2.85%), leakage (2.45%), labeling issue (1.78%), no request (1.60%), un-centrifuged (0.94%), insufficient quantity (0.71%), and clotting (0.27%). In addition, about 11.23% of the rejected samples were a result of a combination of more than one of the above reasons (Fig. 4).

**Table 3:** Reasons for rejection of PHC specimens correlated with the most possible causes and action plan

Reason of rejection	%	Expected underlying cause	Action plan
No sample	68.30%	Phlebotomy process	Phlebotomy training and competency program for nurses
Incorrect tube	6.95%	Phlebotomy process	Phlebotomy training and competency program for nurses
Incorrect sample	2.90%	Phlebotomy process	Phlebotomy training and competency program for nurses
Hemolysis	2.85%	Phlebotomy process Transportation Lab personnel competency on specimen handling	Phlebotomy training and competency program for nurses Training on specimen transportation and spill kit safety for drivers Specimen reception training for laboratory personnel
Leakage	2.45%	Phlebotomy process Transportation Lab personnel competency on specimen handling	Phlebotomy training and competency program for nurses Training on specimen transportation and spill kit safety for drivers Specimen reception training for laboratory personnel
Missing or incorrect label	1.78%	Phlebotomy process	Phlebotomy training and competency program for nurses
No request	1.60%	Physician awareness	Physician awareness on the unified laboratory guide
Un-centrifuged	0.94%	Lab personnel competency on specimen handling	Specimen reception training for laboratory personnel
Insufficient quantity	0.71%	Phlebotomy process	Phlebotomy training and competency program for nurses
Clotted	0.27%	Phlebotomy process Transportation Lab personnel competency on specimen handling	Phlebotomy training and competency program for nurses Training on specimen transportation and spill kit safety for drivers Specimen reception training for laboratory personnel
Other (combination)	11.23%	Phlebotomy process Transportation Lab personnel competency on specimen handling

#### The TAT

**Fig. 5:**
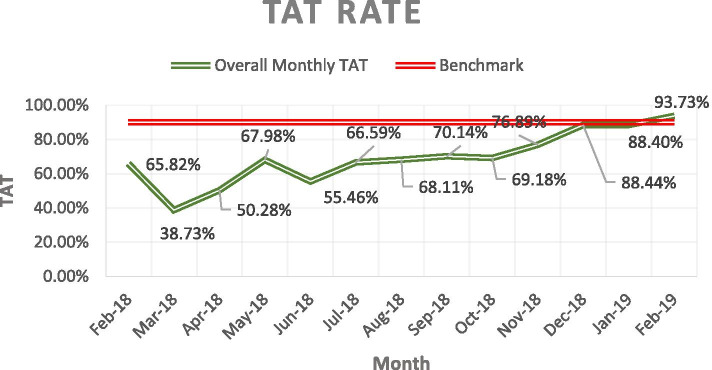
This figure shows the improvement of the TAT rate over the first year. The bench mark for accepted TAT in KFMC Laboratory is ≥ 90% (based on the CAP Guidelines), calculated based on the number of total samples resulted on-time to the number of total samples accepted for analysis

The TAT result was monitored monthly and is presented in Fig. 5. Before electronic connectivity with the PHCs, results were communicated manually through the transportation team, in a sealed package to ensure confidentiality, each time samples were transported. Starting from October 2018, the Laboratory Information System (LIS) system was launched in the first PHC. By February 2019, a total of four out of seven PHCs were using the LIS system for test requests, rejection communication, accessioning, and result reporting.

#### Amendment of Results

Amendment of results reflect the post analytical process of a laboratory, in the whole project time a total of 142/32,692 samples were amended.

## Discussion

With the increasing global aging population [10] and the move toward urbanization [1], the Kingdom of Saudi Arabia launched the NTP with the aim of improving the quality and efficacy of the healthcare system [7]. One of the major initiatives of the NTP was improving the primary healthcare services. Previously, the majority of studies on PHCs within the KSA were cross-sectional studies that focused on patient satisfaction surveys, outbreak investigations, and vaccination campaigns [10, 21]. To our knowledge, this is the first study in KSA that is focusing on the laboratory service and the impact of both the implementations of CBAHI PHC standards and centralization of the laboratory services to an accredited reference laboratory in quality improvement and re-gain of population trust in the PHCs laboratory services, a step towards the implantation of the one of the KSA NTP initiatives.

In February 2018, ten PHCs joined the C2 healthcare cluster based in Riyadh. At that time, some of the laboratory tests were done in-house within the PHC as POCT-based testing or other testing were directed to one of the bigger PHCs if the original PHC had a limited testing menu. In order to clearly define the baseline quality status of laboratory services within the PHCs, an initial decision to centralize and direct the laboratory testing at the KFMC laboratory was made so that the pre-analytical laboratory processes within the PHCs could be adequately assessed. Also, to assess the efficiency of centralization of the laboratory services as a model comparing to having them done within each PHC. The in-house laboratory activities within the centers were limited to three: (1) POCT-based testing, which included urine-based pregnancy testing, urine dipstick, and glucose level testing using the glucometers, (2) Phlebotomy activities, including specimen collection, separation (when needed) and storage, and (3) packaging for transportation, in addition to all daily quality and maintenance activities related to these tasks. Brainstorming sessions between the Executive Administration of Community Health team, PHC directors, laboratory team at KFMC, and family medicine physicians, showed that the laboratory services across the PHCs were not standardized, many equipment were old and out of maintenance contracts, staff competencies were not monitored regularly, there were inconsistent supply issues, the out sourced tests were faced with high rejection and sample loss rates, and in general the PHCs directors were not satisfied with the laboratory services. This lead the agreement among the stakeholders to first define a unified PHC laboratory guide, which included the testing menu based on the best practices clinical guidelines in family medicine, sample requirement, sample storage, and transportation requirement, collection schedule, the transportation of each PHC, transporter information, special requirements, TATs, and contact personnel at KFMC for any queries or emergencies [18].

In any Clinical laboratory, the total testing process of a laboratory (TTS) is divided into three phases: pre-analytical, analytical, and post-analytical [22]. Laboratories should be actively involved in the design, monitor and control of all the process phases [23]. For each phase, a KPI was selected to monitor the quality of the process, help reduce laboratory errors, and ensure patient safety. In addition, the cost of rejection, laboratory personnel time spent in direct and non-direct laboratory tasks, and test request habits were used to define and measure the utilization of laboratory testing [24]. The regular review of these KPI measures helps identify and minimize significant variations and trends. The selection of which KPI's to monitor was left to the laboratory management, provided it included the pre-analytical, analytical, and post-analytical phases of the testing process, with an emphasis on the pre- and post-analytical phases [22, 25, 26].

The initial CBAHI internal mock visits to assess the base line quality against the laboratory section under the CBAHI PHCs standards were done twice a week at each PHC (standards & required EC in Table 1). The initial internal mock assessment visit included nine out of ten centers, one center was temporarily suspended for renovations. Additional two centers were temporarily suspended for renovations later on, so by the end of the first year a total of seven PHCs were functioning and revisited for the follow-up CBAHI internal mock assessment to follow-up with the interventions. However, the populations served by the temporarily suspended PHCs were redirected to the nearest operating PHC to avoid service interruption. We sought to bring each PHC to no less than 80% compliance with the CBAHI laboratory standards following our interventions. The detailed internal mock assessment results were presented above in the results sections. The initial (first) visits to the PHC revealed significant noncompliance on the level of both document review and record evidence throughout the 11 CBAHI PHC standards and 56 sub-standards. To ensure standardized and unified practice and consistency in the service the first action plan was to produce a unified document control system for the PHC based on the national and international guidelines [18–20], then train the laboratory staff and nurses performing POCT on the unified policies and procedures and asses their competencies. The documents created included, a laboratory guide which describes: the test menu, specimen collection, storage, and transportation conditions, TAT, critical result reporting, staff competency programs, inventory management programs, equipment management programs, quality, and safety programs, reagents handling programs, and contacts in the case of emergencies or queries. The documentation and record have been validated and implemented within the system and are subjected to review and update as per the best practices guidelines [18–20].

During the first 13 months, a total of 32,692 samples were sent to KFMC. To measure the pre-analytical performance, the sample rejection rate, sorted by rejection reasons, was monitored. Of the 32,696 samples received during the first year, a total of 2243 samples were rejected, giving a general average rejection rate of 6.86% for the first year (Fig. 3). The baseline rejection rate within the first quarter was of an average of 15.5%, peaking to 21.98% for the third month. The reasons for rejection (Fig. 4) reflected the underlying performance measures which needed to be improved. In this study, the highest reason for rejection (60.30%) was the absence of a sample or an insufficient sample for the requested test. Internationally, insufficient samples usually originated from the difficulty in collecting a sufficient volume of blood from newborns, children, and other challenging patients, such as oncology patients, who require venipuncture procedures that can only be performed with special training and skill [27], however our findings showed that the sample insufficiency reason was related to the staff knowledge of sample requirements and the absence of proper reference documentation related to sample collection (collection manual). When a group of laboratory tests were requested, the peripheral blood container for more than one test may be the same, but the testing site was different. For example, Complete Blood Count (CBC) and Haemoglobin A1c (HbA1c) both require the purple top vacutainer with Ethylenediamine tetraacetic acid (EDTA) anticoagulant. In this case, a single tube for all tests using the same vacutainer was sent from the PHC. Upon arrival at the testing site at KFMC, it would either be sent to a hematology lab for CBC or biochemistry analysis for HbA1c, where the whole sample will be consumed within the automated system. As a result, one of these tests will be completed and reported, while the other will be rejected due to the absence of a sample. This, is one of the reason for rejection (Table 3) that reflected a need to improve staff competencies as well as the presence of a liable documentation and policies to serve as reference for the PHC staff.

It has been extensively documented that most laboratory errors occur in the pre-analytical phase, which is not under the direct control of the testing laboratory [27]. Lay et al. (2014) presented the clotted specimen as one of the higher reasons for rejection and emphasized the fact that depending on the reason for rejection, proper interventions can be designed [28]. Our results showed that in the pre-analytical phase, sample collection, and handling competencies should be of the utmost focus in standardizing phlebotomy and laboratory practices. Other underlying causes of persisting rejection rates, besides competency, included, also the lack of issues related to instruments and supply. For example, the lack of centrifuge setups, frequent equipment breakdowns in some PHCs, supply delivery problems, laboratory staff shortage, and vacations in some of the PHCs, which may have compromised the specimen integrity and have been documented in the literature [24, 28]. The rejection rate, sorted by reasons, was an integral KPI that was monitored and communicated daily to the PHCs through the LIS system (if available) or through email.

In general, TAT, critical result reporting, and amendment of results are three crucial KPIs covering the analytical and post-analytical phases of the TTS [22]. In this project we focused on the TAT as the KPI to monitor the analytical phase of the whole TTS and it will give us a clear indication on how effective the centralization of all tests to a reference hospital is. Physicians need an accurate and timely result to make correct clinical decisions [24, 29], the TAT benchmark states that at least 90% of the tested sample must be reported within the expected TAT (which differs from test to test), this cycle was done manually at the beginning of this PHC pilot project as there was a no existing LIS within the PHCs which lead to an unacceptably low TAT (Fig. 5). From a physician’s perspective, the methods suggested to improve the TAT may include pneumatic tubing systems for large institutes, satellite laboratories to cover large geographic areas, the implementation of point-of-care testing, and an investment in an efficient computer technology with an LIS system that provides traceability for the entire testing process [30]. One of the five strategies proposed for laboratories to reach reaching their full potentials at an ACO institute is about electronic connectivity that enables physicians to easily request and access laboratory tests as they need [3, 12]. In this pilot project, the introduction of the LIS system, full-cycle testing, and launching within the PHC were done gradually with one PHC at a time. Our result showed a significant improvement of TAT from an average of 50.80% in the first 7 months to an average of 81.13% in the last 6 months, reaching 93.73% by the first month of the second year of service, which is a rate that falls within the benchmark of routine tests TAT and exceeding our first-year set target of 80% [18–20].

The amendment of a verified and sent result is an indication that reflect the post-analytical phase in a TTS. A total of 142 results were amended, making the amendment rate of 0.4%, which also reached our first-year target. In addition to the above monitored KPI's, the critical result as a KPI was a necessity to urgently prepare a policy for reporting and escalation, to be part of the PHC KPI's system once finalized. An algorithm was defined, with a contact number for each PHC, for the critical result to be delivered to the caregiver, with a "read-back" and documentation of the whole process, as per the best practice national and international standards [19]. In order to avoid unreported critical results, the policy defined an escalation process that was activated within 5 min of any failure to provide the critical results to the defined caregiver in each PHC [26].

From a cost and best-utilization perspective, when a sample is rejected, all the pre-analytical effort and cost was considered lost, by defining and calculating the pre-analytical cost per sample (overviewed in the methodology section), and measuring the improvement in the rejection rate between the highest rejection rate month, with a total of 548 samples (24%), and first month of the second year with the lowest rejection rate, with a total of 88 samples only (1.7%). By the last month of this project, the monthly cost of rejection was about 70% lower comparing to the month with highest rejection rate, reflects the savings garnered by improving the quality of this particular KPI. Another utilization measure was the use of laboratory personnel time for direct and indirect laboratory tasks. This was focusing only on two categories of staff and from a laboratory tasks prospective only: these are the nurses and the laboratory personnel. Following the centralization of the laboratory services to a reference hospital; the duties of the laboratory personnel was limited to the tasks when there is sample collection within the PHCs. Usually, an average PHC collects samples 3 days per week and has a single morning session of around 3 h of direct and indirect lab work. By calculating the monthly working hours at 140 h/month, the lab personnel performed lab-related tasks for 26% of their monthly working hours only. This means that 76% of their time is spent on non-lab tasks, which could be administration related. Therefore, re-assessment of staff time utilization is essential as a future phase of this project.

Competency assessment programs (CA) are used within the laboratories to determine personnel’s ability to correctly apply their knowledge, skill, and experience to their laboratory duties. The main purpose of a laboratory competency assessment program is to define and improve any incompetence in personnel performance, which may compromise patient services and patient safety [31]. Following the initial phase of the baseline pre-analytical laboratory services assessment using CBAHI standards, one of the major issues was related to in-consistent work practice and variation in knowledge and skills of the staff. The main intervention was to design and implement the PHC staff competency assessment program. This program was modified from the current CA testing used in KFMC and based on the CAP and CLIA standard requirements. The training program described in the methodology section commenced in the third month of this pilot project, was ongoing, and focused on the pre-analytical phase, since the majority of the findings were related to the pre-analytical phase and consistent practice. This included phlebotomy training in two parts, the first part was conducted off-site within the KFMC phlebotomy section to demonstrate a model of laboratory service and provide hands-on experience under the supervision of the fully trained and qualified phlebotomist, who graduated from the Saudi Commission for Health Specialties (SCFHS) approved phlebotomy training program. This was followed by on-site training of each PHC at their respective sites which helped to establish the phlebotomy unit per the best practices guidelines and CBAHI and CAP standards. The outcomes of these interventions are reflected in the decreased rejection rates of quarters 2,3 &4 respectively 5.95% for Q2, 7.43% for Q3, 5.57% for Q4. By the first month of the second year the rejection rate went down to 3.92%. Although the 3.92% rejection rate did not reach the 0.4–0.6% benchmark, however it exceeded our first-year target of 7% rejection rate.

Coordinated high-quality and cost-effective healthcare is the core purpose of moving towards an ACO systems [12]. Five strategies have been outlined for laboratory services to assist healthcare organizations in reaching their full potentials in an ACO model: (1) outreach services and programs, (2) electronic connectivity, (3) lean processes, (4) utilization management, and (5) alignment with the bigger picture [3, 12]. The model followed in this pilot project demonstrated how implementing some of these strategies contributed to the cost-effective improvements. Clinicians are seeking faster test results however, to establish an independent laboratory for each PHC to cover the laboratory needs for its population was not cost-effective. In order for it to be so, each PHC must have had a high population to cover the system prices, operating staff, proficiency testing, and accreditation cost. Moreover, increasing the POCT testing menu was not the most optimal solution due to the performance precision of some testing systems as well as the cost per test [32]. It is estimated that 25% of laboratory tests were unnecessarily ordered which leads to a huge potential waste, moreover, there might be a huge variability in the laboratory tests ordered by the different physicians [24]. In this model having an out-reach program for the laboratory services with pre-defined testing menu that was based on the best practices was found to be more efficient and gave better quality and utilization of the services as shown by the improved KPIs result as well as the increased demands over time. Interventions related to closing the communication gap between PHC physicians and the KFMC lab and the introduction of the LIS system made the requesting and result reception a lean process [33]. Communicating the potentials of this piloted model and presenting the KPI results to the PHC directors aided in securing the proper support for going forward with this model, which was reflected in the gradual increase of the samples tested from month to month (Fig. 3).

Like any study there are areas of improvement and possible future direction of this pilot project, these may include focusing on improving the time periods between obtaining and receiving specimens at the testing laboratory. One suggestion is to implement a satellite laboratory within an "urgent care PHC" to serve the urgency scope of some of the PHC as well as decrease the geographic distance, that an urgent sample has to travel to reach KFMC. This may also decrease specimen rejections due to specimen integrity or deterioration of analytes, this may overcome urgent specimen transportation issues, that we may face in this current model [32]. Moreover, the continuation of establishment of an LIS system across PHCs will help in minimizes labeling errors, unifies test request practices, serves as a communication platform for results and rejection, provides data extraction for quality and performance analysis, and is standardized and protected [24, 33, 34]. Lastly the, utilization of personnel time is an area to focus on in the future as it may positively impact the cost of services.

## Conclusions

The centralization of the majority of laboratory services provided at the PHCs and directing it to an accredited laboratory strengthened the standardization initiatives of the laboratory practice within the 10 piloted PHCs, improved quality, and decreased costs (for example the analytical cost, PT enrolment for each PHC, and the pre-analytical cost resulting from rejection), provided that there was an implemented POCT program serving the scope of each PHC. Staff competency serves as the human power behind the machines, so implementing a staff CT programs are an integral part of any patient-safe services. Moreover, having these training programs in collaboration with a reference institute led to better outcomes. Having a unifying documentations system (policies and procedures) is crucial to standardize the practice within PHCs and unify knowledge and skills of the working staff to achieve better quality for the patient services. Electronic connectivity led to immediate positive impact on pre-analytical, analytical & post-analytical phases of laboratory testing, and facilitated more lean testing-result cycle with in the connected laboratories as shown in the TAT rate KPI. A proper definition of KPIs, and the close monitoring of them is effective in assessing the quality status of the PHCs and defining the proper interventions and corrective actions in a timely manner. We consider the above model effective for urban cities, where an accredited central laboratory is approachable for PHCs.

## Data Availability

Available.

## Abbreviations

ACO: Accountable Care Organizations
AEACH: Associate Executive Administration of Community Health
AEAMA: Associate Executive Administration of Medical Administrations
C2: Second Central Healthcare Cluster
CAP: College of American Pathologists
CBAHI: Central Board for Accreditation of Health Institutes
CLIA: Clinical Laboratory Improvement Amendments
EC: Evidence of compliances
ESR: Essential Safety Requirement
FOCUS: Find, Organize, Clarify, Understand, & Select.
KFMC: King Fahad Medical City
KPI: Key performance indicators
KSA: Kingdom of Saudi Arabia
MOH: Ministry of Health
NTP: National Transformation Program
PCLMA: Pathology and Clinical Laboratory Medicine Administration
PDCA: Plan, Do, Check, and Act,
PHC: Primary healthcare centers
POCT: Point of Care Testing
TAT: Turn-around Time
TTS: Total Testing
WHO: World Health Organization
